# 

**Published:** 1858-08

**Authors:** 


### CONTENTS.

| | |
|---|---|
| ORIGINAL COMMUNICATIONS— | PAGE. |
| Physiology, Pathology and Therapeutics of Muscular Exercise. By W. H. Byford, M.D | 357 |
| Case of Extraction of the Bones of a Fœtus. By D. W. Young, M.D.... | 383 |
| Case of Fracture of the Lower Jaw at its Neck. By M. O. Heydock, M. D | 387 |
| Case of Disease of the Brain. By B. Woodward, M.D | 391 |
| Examine your Medicines. By Jno T. Plummer | 394 |
| Translations—By Dr. Byford— | |
| A Case of Natrophagie | 396 |
| BOOK AND PAMPHLET NOTICES— | |
| Transactions of the Twenty-ninth Annual Meeting of the Tennessee State Medical Society | 398 |
| EDITORIAL— | |
| Reports of the Sanitary Committee to the Cook Co. Medical Society...... | 402 |
| Apology and Bills | 414 |
| Rush Medical College | 414 |

